# *Leptotrombidium imphalum* Chiggers as Vector for Scrub Typhus in Human Settlements, India, 2022–2023

**DOI:** 10.3201/eid3202.251170

**Published:** 2026-02

**Authors:** Carol S. Devamani, Neal Alexander, Rawadee Kumlert, Benjamin L. Makepeace, Serge Morand, Mary Cameron, Alexandr A. Stekolnikov, Winsley Rose, Daniel Chandramohan, Punam Mangtani, Kundavaram P.P. Abhilash, Wolf-Peter Schmidt

**Affiliations:** Christian Medical College, Vellore, India (C.S. Devamani, W. Rose, K.P.P. Abhilash); London School of Hygiene and Tropical Medicine, London, UK (N. Alexander, M. Cameron, D. Chandramohan, P. Mangtani, W.-P. Schmidt); Ministry of Public Health, Nonthaburi, Thailand (R. Kumlert); University of Liverpool, Liverpool, UK (B.L. Makepeace); Centre National de la Recherche Scientifique, France–Kasetsart University and Mahidol University, Bangkok, Thailand (S. Morand); Zoological Institute of the Russian Academy of Sciences, St. Petersburg, Russia (A.A. Stekolnikov)

**Keywords:** scrub typhus, bacteria, Leptotrombidium imphalum, Trombiculidae, vector-borne infections, India

## Abstract

Scrub typhus is a common bacterial infection in many parts of Asia. The causative agent, *Orientia tsutsugamushi*, is transmitted by trombiculid mite (chigger) larvae that require small mammals as maintaining hosts. We studied the prevalence of *O. tsutsugamushi* infection in mites and small mammals in villages and land surrounding them in South India to determine high-risk settings. We identified 12,431 mite larvae on 883 small mammals, 32% of which were bandicoot rat*s,* 31% black rats*,* and 31% Asian house shrews. *Leptotrombidium imphalum* was the most common mite species and the only species associated with *O. tsutsugamushi* infection (prevalence 3.6%). *Orientia* infection increased with mite population size on a host. Host numbers, the *L. imphalum* index, and the prevalence of *Orientia* infection in chiggers were considerably higher within human settlements than in surrounding fields, suggesting that most human scrub typhus infection occurs inside villages rather than during agricultural work.

Scrub typhus is caused by intracellular bacteria of the genus *Orientia* (family Rickettsiaceae, order Rickettsiales) (*1*). Scrub typhus, caused by *O. tsutsugamushi*, occurs predominantly in South Asia, East Asia, and Southeast Asia (*2*). Severe infection is characterized by acute respiratory distress syndrome, shock, renal failure, and meningoencephalitis (*3*). *Orientia* bacteria are transmitted by the bite of trombiculid mite larvae (chiggers) (*4*) that use small mammals such as rodents and shrews as their primary maintaining hosts (*4*). Humans are regarded as accidental hosts. The India subcontinent is a region marked by a high occurrence of scrub typhus, accounting for up to 35% of hospital admissions for fever (*3*,*5*–*9*).

Groups regarded at risk for scrub typhus, including farmers and military personnel, are thought to acquire infected chiggers in agricultural fields and within disturbed ecosystems such as forest edges (*4*). However, agricultural and other outdoor activities were only weakly associated with scrub typhus in rural settings in South India (*10*,*11*). That finding was confirmed in a cohort study on risk factors for scrub typhus in the state of Tamil Nadu, in which agricultural activities, taking animals for grazing, firewood collection, and open defecation did not increase the risk for scrub typhus (*12*). Furthermore, persons residing at the edge of a village were not at a higher risk than those in the village center. To better understand scrub typhus transmission in South India, we trapped small mammals in highly endemic villages to estimate parameters of scrub typhus ecology.

## Methods

We conducted our study in the context of a human cohort study on scrub typhus in 32,279 persons of all ages living in 37 scrub typhus–endemic villages in Vellore and Ranipet, 2 districts of Tamil Nadu (*13*). Study villages had an average size of ≈225 households; mean household size was 4.0. Approximately 55% of the population practiced part-time or full-time agriculture. The Institutional Animal Ethics Committee of the Christian Medical College Vellore (Ref 09/2019) and the Animal Welfare and Ethical Review Board of the London School of Hygiene and Tropical Medicine (Ref 2019-10) approved the study. We followed institutional and national (India and United Kingdom) standards of animal care and use.

### Trapping of Small Mammals

For logistical reasons, we included in this study 25 of the 37 villages in the human cohort study that were within 45 minutes driving distance from the study center. We conducted trapping continuously during August 2022–September 2023, visiting each of the 25 villages twice. We aimed to visit each village once in the rainy season (approximately June–December) and once in the dry season (January–May). At each village visit, we set traps for 4 consecutive nights, Mondays through Thursdays, choosing different trap locations at each of the 2 visits. We used locally available single-capture cage traps, 27 cm × 15 cm × 12.5 cm, that contained a bowl of water and coconut and peanut butter as bait. We set traps in 3 different habitats: village center, village edge, and fields surrounding the village (Appendix Figure 1). We used Google Maps (https://www.google.com/maps) to visualize geographic village centers with households closest to the center chosen for trapping within the household compound but outside the house. We chose the 2 edges of the village nearest the village center and selected households located at the edge for trapping within the compound. We allowed for some flexibility in selecting the village edge to account for unevenly shaped village borders and access to surrounding fields. 

We set traps in the field at >50 m from the village edge, measured from the houses on the village edge selected for trapping; we determined locations of the traps by the presence of trees to which traps were chained to reduce loss. Because success was highest in the center and lowest in the field, we set 4 traps in the center, 6 traps at the edge, and 10 traps in the fields to achieve similar catches in all 3 habitats. Traps were open during the night and closed during the day to reduce catching nontarget animals. Each morning, we inspected traps for catches and brought captured target animals (small mammals) to the study center inside the cage in an air-conditioned vehicle. At the study center, we euthanized the animals by carbon dioxide inhalation, then measured body, tail, hindfoot, and ear length for species identification, using published guidance (*14*). We inspected animals for any visible ectoparasites. We clipped their ears at the base and stored them in absolute ethanol at −70°C. For animals on which we found mite colonies on their hindlegs, we clipped and stored the hindlegs. We dissected the animals and removed spleen tissue for immediate storage at −70°C without ethanol (*15*).

### Chigger Enumeration and Identification

We examined ears and legs of each animal under a stereomicroscope (StereoBlue EVO; Euromex, https://www.euromex.com). We enumerated chiggers on ears and legs separately; because the overall number found was large, we used samples of chiggers for species identification and molecular analysis. We selected up to 10 chiggers from each ear or leg for mounting on microscopy slides, using Hoyer medium for morphologic identification, and placed up to 20 in an Eppendorf tube in absolute ethanol for molecular analysis. To ensure that chigger species on slides and in Eppendorf tubes were approximately comparable, we alternately chose chiggers for slide mounting or molecular analysis in groups of up to 5. In shrews, we found more chiggers on hindlegs than ears. In those cases, we used chiggers from legs for mounting and for PCR; we counted chiggers found on ears but did not further process them.

We identified chiggers under a light microscope (iScope; Euromex) using a key for chigger mites in India (*16*). We confirmed identification of species from the genus *Leptotrombidium* and related genera using published keys (*17*,*18*).

### DNA Extraction and Molecular Analysis

We extracted DNA from chiggers and spleen tissue using a DNeasy Blood & Tissue Kit (QIAGEN, https://www.qiagen.com). We used quantitative PCR to detect *O. tsutsugamushi* targeting the 47-kDa protein gene as previously described (*19*). For each morphologically identified rodent species, we selected 2 representative specimens for molecular barcoding (Appendix).

### Statistical Analysis

We calculated the proportion of small mammals infested with chiggers by mammal and chigger species, as well as the chigger index (i.e., the mean number of chiggers per host) (*4*). To account for the sampling process of selecting a subset of chiggers for identification, we calculated the chigger index for different mammal and chigger species by extrapolation from the species composition observed for different locations on a host (each of 2 ears and hindlegs). We averaged the proportions of each chigger species from different locations on the same host and weighted the averages by the total number of chiggers on that location (mounted or not). We then applied the weighted average proportions to the total number of chiggers found on that host to arrive at the species-specific chigger index.

**Figure 5:**
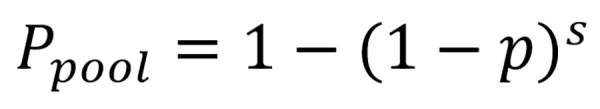
Pool size and pool positivity formula using complementary log-log models.

Similarly, we estimated the number of chiggers of each species in a pool undergoing PCR from the proportion of microscopically identified chiggers of that species at the same location from which chiggers for the PCR pool were selected. We calculated the association between the estimated number of chiggers of 1 species in the pool and *O. tsutsugamushi* PCR positivity of the same pool using generalized linear models (binomial family, identity link). We explored effect modification by habitat as a 3-category variable (village center, edge, field) using likelihood ratio tests. We estimated the prevalence of *O. tsutsugamushi* in individual chiggers, *p*, on the basis of pool size and pool positivity using complementary log-log models based on the formula (Figure 5) (*20*) where *P_pool_* is the proportion of pools that are positive for *O. tsutsugamushi* and *s* is the pool size (Appendix).

We estimated the association between the total number of *Leptotrombidium imphalum* chiggers on a host and the probability of *O. tsutsugamushi* PCR positivity of the chigger pool from the same host using logistic regression with PCR positivity as outcome and the estimated number of chiggers (chigger index) as explanatory variable, modeled as cubic splines with knots at 0, 100, and 200 chiggers. We resolved a pronounced right skew in the chigger index by applying the 4th root, and we adjusted for pool size because small mammals with a high chigger index tended to have larger pool sizes. We explored the association between chigger index and the probability of the spleen sample being PCR positive for *O. tsutsugamushi*. We expressed clustering of trap success and *Orientia* infection prevalence within villages as the intraclass correlation coefficient (ICC).

## Results

### Small Mammal Trapping by Habitat and Season

We trapped 883 small mammals during 9,496 trap nights, an overall trap success of 9.3% (Table 1). Trap success was 20.3% in the village center (314 during 1,545 trap nights), 16.6% on the village edge (297/1,794) and 4.4% in the fields (272/6,152). There was some degree of clustering of the overall trap success within villages (ICC = 0.011; p = 0.001).

**Table 1:** Small mammal distribution by habitat in study of *Leptotrombidium imphalum* chiggers as vector for scrub typhus in human settlements, India, 2022–2023

Animal	No. (%) animals			
	Village center	Village edge	Field	Total
Greater bandicoot rat, *Bandicota indica*	156 (49.4)	105 (35.4)	23 (8.5)	284 (32.2)
Black rat, *Rattus rattus*	79 (25.0)	85 (28.6)	113 (41.9)	277 (31.4)
Asian house shrew, *Suncus murinus*	80 (25.3)	102 (34.3)	91 (33.7)	273 (30.9)
Mouse, *Mus* spp.	1 (0.3)	0	21 (7.8)	22 (2.5)
Three-striped Indian palm squirrel, *Funambulus palmarum*	0	2 (0.7)	12 (4.4)	14 (1.6)
Indian gerbil, *Tatera indica*	0	3 (1.0)	9 (3.3)	12 (1.4)
Asian gray mongoose, *Urva edwardsii*	0	0	1 (0.4)	1 (0.1)
Total	316 (100.0)	297 (100.0)	270 (100.0)	883 (100.0)

The greater bandicoot rat (*Bandicota indica*), the black rat (*Rattus rattus*), and the Asian house shrew (*Suncus murinus*) accounted for most of the trapped mammals, followed by mouse species (*Mus* spp.), three-striped Indian palm squirrel (*Funambulus palmarum*), Indian gerbil (*Tatera indica*), and Asian gray mongoose (*Urva edwardsii*). The bandicoot rats (GenBank accession no. PV918832) predominantly displayed an unusual tail to head/body ratio >1 (Appendix Figure 3). One mouse specimen (GenBank accession no. PV915568) showed 99.7% sequence identity with *M. saxicola* (GenBank accession no. MN964116). A further *Mus* specimen (accession no. PV915577) showed 98.3% identity with *M. terricolor* (accession no. KY018920). Greater bandicoot rats predominated in human habitats, whereas black rats, Indian gerbils, mice, and squirrels were more commonly trapped in the fields (Table 1). Asian house shrews were common in all 3 habitats.

**Figure 1:**
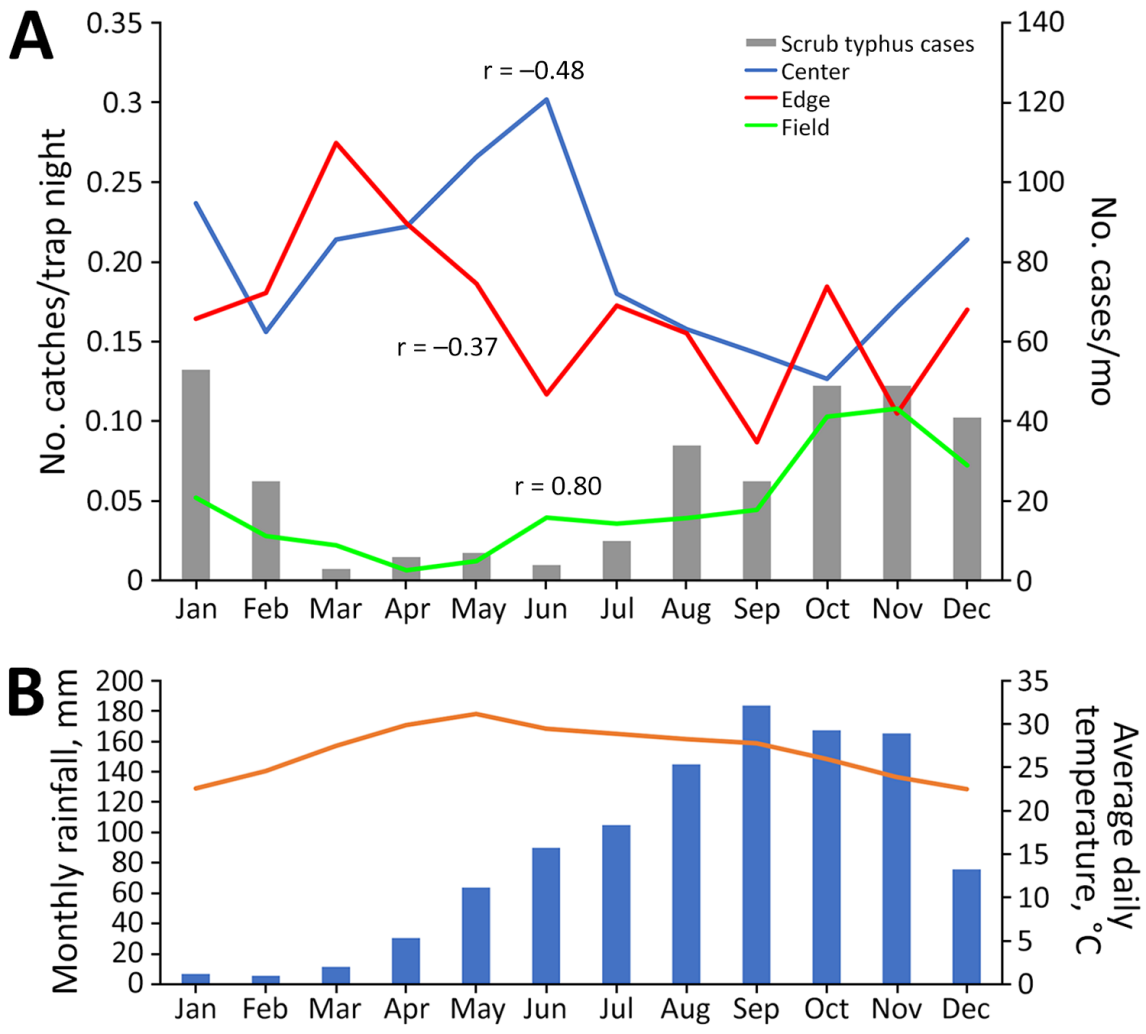
Correlation of trapping success with seasonal characteristics in study of *Leptotrombidium imphalum* chiggers as vector for scrub typhus in human settlements, India, 2022–2023. A) Monthly small mammal catches per trap night, by habitat (center of village, edge of village, or field outside village), compared with numbers of monthly human scrub typhus cases. Case numbers adapted from Devamani et al. (*13*). B) Monthly rainfall (blue bars) and average daily temperature (orange line) 1991–2021. Source: India Meteorological Department (https://mausam.imd.gov.in). Scales for the y-axes differ substantially to underscore patterns but do not permit direct comparisons.

In the fields, small mammal trapping showed pronounced seasonal variation with a low in the dry season and a high in October and November (Figure 1); we observed strong correlation between trappings and monthly human scrub typhus cases (r = 0.80). Trappings in village edge (r = −0.48) and center (r = −0.37) tended to be inversely correlated with scrub typhus cases.

### Chigger Species by Host, Habitat, and Season

We collected 90,377 chiggers; of those, we identified 12,431 morphologically under the microscope and placed 10,740 in 759 pools for PCR analysis. *L. imphalum* was the most common chigger species, followed by *Ericotrombidium bhattipadense* and *Schoengastiella ligula* (Table 2). The *L. imphalum* indices were highest on Indian gerbils and house shrews. Otherwise, *L. imphalum* chiggers did not show a strong host preference, whereas *E. bhattipadense* and *S. ligula* chiggers had a strong host preference for *B. indica* rats. *Trombicula hypodermata* chiggers were found mainly on shrews, whereas *Microtrombicula kajutekrii* was mostly on black rats and *Schoengastia tuberculata* on bandicoot rats. One species of *Hypotrombidium* chiggers and 1 species of *Walchia* chiggers could not be identified to species level (Appendix Figures 4–7).

**Table 2:** Chiggers identified by host species in study of *Leptotrombidium imphalum* chiggers as vector for scrub typhus in human settlements, India, 2022–2023*

Chigger species	Black rat,* *n = 277	Greater bandicoot rat*, *n = 284	Indian gerbil, n = 12	Mouse, n = 22	Three-striped Indian palm squirrel, n = 14	Asian house shrew*, *n = 273	Asian gray mongoose*, *n = 1	Total, n = 883
*L. imphalum*	769 (48.7)	967 (59.9)	80 (75.0)	6 (13.6)	20 (35.7)	2,470 (75.1)	7 (100.0)	4,319 (59.8)
Chigger index (SD)	11.7 (26.5)	34.3 (55.8)	59.9 (91.1)	0.9 (3.3)	3.7 (6.8)	53.9 (74.4)	14.2 (NA)	32.3 (58)
*Leptotrombidium* sp.	2 (0.7)	0	0	0	1 (7.1)	1 (0.4)	0	4 (0.5)
Chigger index (SD)	0.04 (0.5)	0	0	0	0.15 (0.5)	0.01 (0.2)	0	0.02 (0.3)
*Ericotrombidium bhattipadense*	1,098 (63.2)	2,348 (90.1)	4 (16.7)	18 (4.5)	25 (57.1)	665 (51.3)	1 (100.0)	4,159 (66.0)
Chigger index (SD)	13.8 (24.4)	83 (85.3)	0.7 (1.6)	2.4 (11.3)	4.7 (6.2)	12.9 (30.1)	2.8 (NA)	35.2 (62.4)
*Hypotrombidium* sp.	360 (27.1)	134 (18.7)	8 (16.7)	3 (9.1)	122 (78.6)	5 (0.7)	0	632 (16.4)
Chigger index (SD)	6 (21.8)	5.2 (17.7)	1.4 (3.8)	0.3 (1)	37.7 (50.5)	0.1 (0.8)	0	4.2 (17.7)
*Ericotrombidium* or *Hypotrombidium* sp.	13 (1.4)	8 (1.4)	0	0	0	0	0	21 (0.9)
Chigger index (SD)	0.2 (2.1)	0.2 (2.8)	0	0	0	0	NA	0.1 (2)
*Trombicula hypodermata*	1 (0.4)	3 (1.1)	0	0	0	165 (19.4)	0	169 (6.5)
Chigger index (SD)	0.02 (0.3)	0.1 (0.8)	0	0	0	2.4 (6.6)	NA	0.8 (3.9)
*Microtrombicula kajutekrii*	519 (27.4)	2 (0.4)	0	1 (4.5)	0	0	0	522 (8.8)
Chigger index (SD)	5.7 (17.2)	0.01 (0.1)	0	0.05 (0.2)	0	0	NA	1.8 (10)
*Schoengastia tuberculata*	9 (1.8)	33 (5.3)	0	0	0	0	0	42 (2.3)
Chigger index (SD)	0.1 (1.4)	0.9 (4.5)	0	0	0	0	NA	0.3 (2.7)
*Schoengastiella ligula*	271 (22.7)	1,826 (76.4)	37 (50.0)	2 (4.5)	0	12 (2.6)	0	2,148 (33.3)
Chigger index (SD)	3.4 (13.7)	71.9 (112.1)	7.4 (14.6)	0.3 (1.5)	0	0.1 (1)	NA	24.3 (71.9)
*S. liota*	0	0	2 (16.7)	0	0	6 (1.1)	0	8 (0.6)
Chigger index (SD)	0	0	0.45 (1.1)	0	0	0.03 (0.3)	NA	0.02 (0.2)
*S. punctata*	1 (0.4)	0	0	0	0	18 (4.4)	0	19 (1.5)
Chigger index (SD)	0.04 (0.6)	0	0	0	0	0.3 (1.9)	NA	0.1 (1.1)
*S. ralagea*	7 (1.1)	13 (4.2)	0	0	0	173 (15.4)	0	193 (6.5)
Chigger index (SD)	0 (0.4)	0.7 (4)	0	0	0	6.6 (45.7)	NA	2.3 (25.6)
*S. argalea*	0	0	1 (8.3)	0	0	3 (0.7)	0	4 (0.3)
Chigger index (SD)	0	0	0.2 (0.7)	0	0	0.04 (0.6)	NA	0 (0.3)
*S. ceylonica*	20 (1.1)	1 (0.4)	5 (8.3)	53 (40.9)	0	0	0	79 (1.6)
Chigger index (SD)	0.1 (1.7)	0.03 (0.6)	1.8 (6.3)	6.2 (17.3)	0	0	NA	0.2 (3.1)
*S. bengalensis*	0	1 (0.4)	0	0	0	23 (5.9)	0	24 (1.9)
Chigger index (SD)	0	0.01 (0.2)	0	0	0	0.23 (1.2)	NA	0.07 (0.7)
*S. singularis*	0	0	40 (41.7)	0	0	0	0	40 (0.6)
Chigger index (SD)	0	0	28.8 (51.8)	0	0	0	NA	0.4 (6.7)
*Walchia* sp.	0	0	0	8 (18.2)	0	37 (9.2)	0	45 (3.3)
Chigger index (SD)	0	0	0	0.8 (2.2)	0	0.3 (1.7)	NA	0.1 (1)
*Gahrliepia khandalaensis*	0	1 (0.4)	0	0	0	2 (0.4)	0	3 (0.2)
Chigger index (SD)	0	0.01 (0.1)	0	0	0	0.04 (0.6)	NA	0.01 (0.3)
Total	3,070 (92.1)	5,337 (99.3)	177 (100.0)	91 (63.6)	168 (92.9)	3,580 (91.2)	8 (100.0)	12,431 (93.5)
Chigger index (SD)	41.4 (50.2)	196.4 (148.3)	100.7 (106.4)	11 (22.6)	46.2 (49.5)	77 (98.1)	17 (NA)	102.4 (124.4)

*Chigger species and total values are no. chiggers (% infestation). Chigger index is mean number of chiggers per host. NA, not applicable.

**Figure 2:**
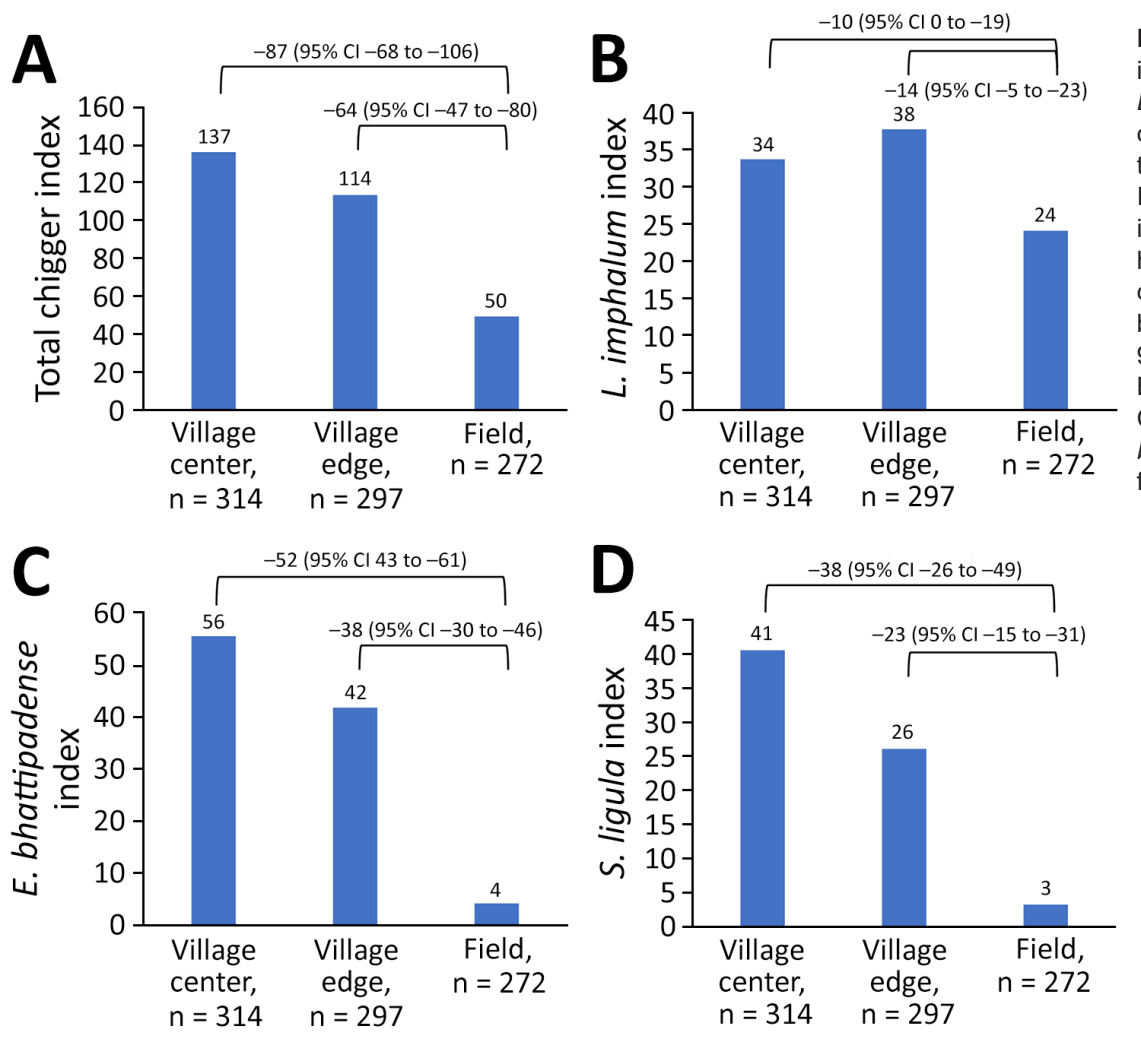
Differences in chigger index by habitat in study of *Leptotrombidium imphalum* chiggers as vector for scrub typhus in human settlements, India, 2022–2023. Chigger index is mean number of chiggers per host. Values above bars indicate chigger index; values above brackets indicate differences and 95% CIs. A) Total chigger index. B) Index for *L. imphalum* chiggers. C) Index for *Ericotrombidium* *bhattipadense* chiggers*.* D) Index for *Schoengastiella* *ligula* chiggers*.*

**Figure 3:**
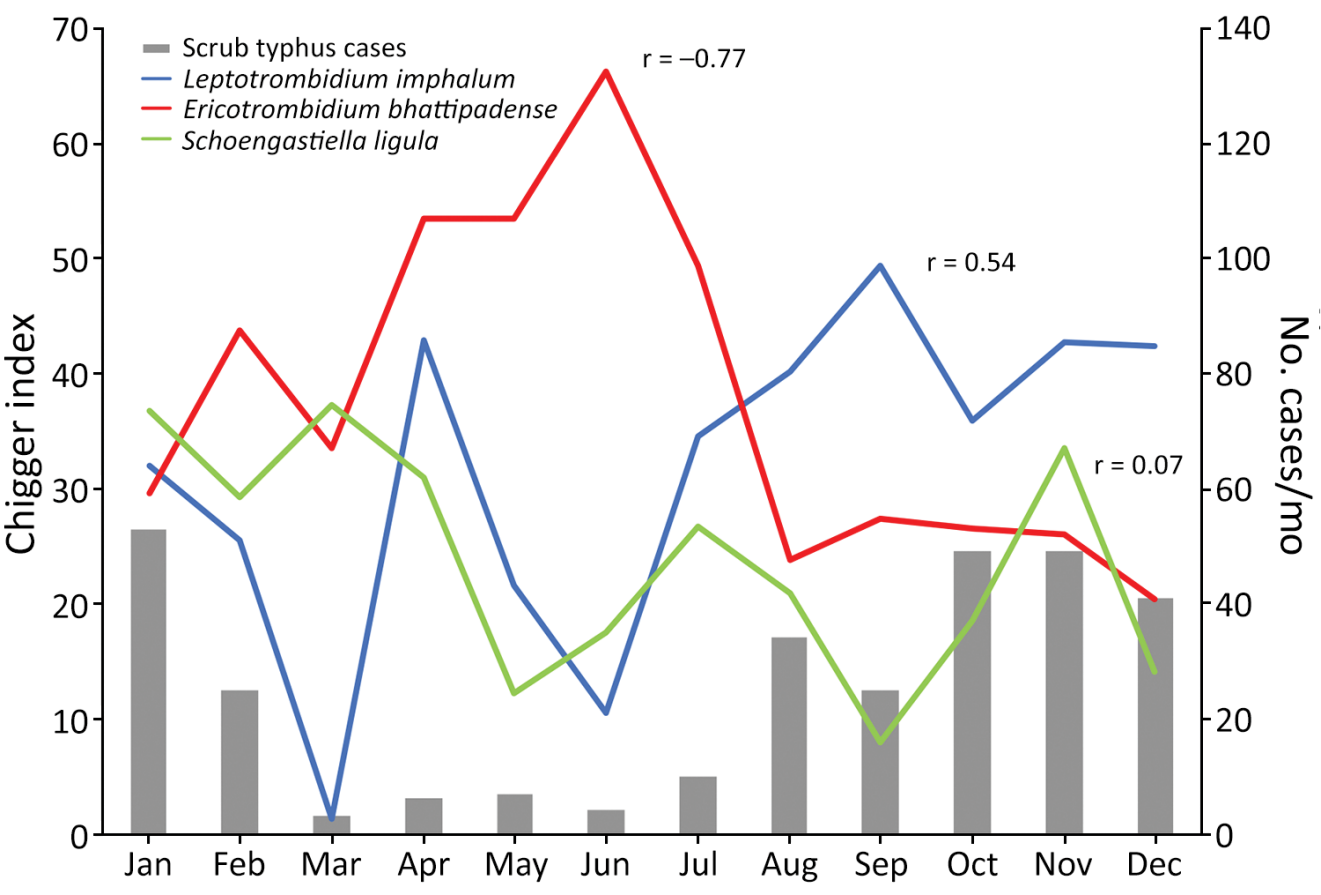
Chigger index by month in study of *Leptotrombidium imphalum* chiggers as vector for scrub typhus in human settlements, India, 2022–2023. Chigger index is mean number of chiggers per host; r is correlation coefficient between chigger index for each species and monthly human scrub typhus cases. Case numbers adapted from Devamani et al. (*13*).

The total chigger index, as well as the indices for *L. imphalum*, *E. bhattipadense*, and *S. ligula* chiggers, were far higher in the village center and edge than in the fields (Figure 2). The *L. imphalum* index correlated moderately with monthly scrub typhus cases (r = 0.51) (Figure 3), whereas the *E. bhattipadense* index showed a strong negative correlation with scrub typhus, peaking in the hot months of April–July (r = −0.78). The *S. ligula* index showed no association (r = 0.08).

### *O. tsutsugamushi* Infection by Chigger and Host Species

By real-time PCR, 122/759 pools (16.1%) tested positive for *O. tsutsugamushi* (Table 3). The coefficients indicate the change in the prevalence of positivity by PCR as a function of the expected number of chiggers of each species per pool, the latter extrapolated from the proportion of each chigger species among specimens examined by microscopy from the same ear or leg. Each additional *L. imphalum* chigger expected to be in a pool was associated with a 2.2% percentage point increase in the PCR pool positivity. By contrast, an increase in the number of most other chigger species was associated with a decreased probability of PCR positivity. *Schoengastia tuberculata* was associated with an increased PCR positivity, but with a wide confidence interval. *L. imphalum* chiggers were found concurrent with *S. tuberculata* in 9/13 locations where *S. tuberculata* was found on slides.

**Table 3:** Molecular detection of *Orientia tsutsugamushi* in chigger mite pools in study of *Leptotrombidium imphalum* chiggers as vector for scrub typhus in human settlements, India, 2022–2023*

Chigger species	No. pools expected to contain species, N = 759	Coefficient (95% CI)
*L. imphalum*	415	2.2% (1.7%–2.6%)
*Ericotrombidium bhattipadense*	470	−0.3% (−0.7% to 0.1%)
*Hypotrombidium* sp.	103	−0.4% (−1.2% to 0.4%)
*Schoengastia tuberculata*	13	2.4% (−2.9% to 7.7%)
*Schoengastiella ligula*	231	−0.5% (−0.9% to 0.0%)
*Schoengastiella ralagea*	41	0.4% (−1.5% to 2.2%)
Any *Schoengastiella *sp*.*	290	−0.5% (−0.9% to 0.1%)
*Microtrombicula kajutekrii*	65	−0.9% (−2.3% to 0.5%)
*Trombicula hypodermata*	41	−0.3% (−2.3% to 1.7%)
*Walchia* sp.	12	NA

*Coefficient is the absolute change in probability of PCR positivity of a pool per additional chigger of a given species. NA, not applicable.

The percentage point increase in PCR positivity with each additional expected *L. imphalum* chigger was higher in the village center (+2.8%, 95% CI 2.0%–3.5%) and edge (+2.4, 95% CI 1.6%–3.1%) than in the field (+1.2%, 95% CI 0.5%–2.0%), with evidence for effect modification (p = 0.018). The overall prevalence of infection with *O. tsutsugamushi* among individual *L. imphalum* chiggers was 3.6%. The prevalence was 2.3 times higher in the village center (4.5%; prevalence ratio 2.3, 95% CI 1.3–4.1) and 2.2 times higher in the village edge (4.2%; prevalence ratio 2.2, 95% CI 1.2–3.8) than in the fields (1.9%). The prevalence of infection in chigger pools was highest in those from house shrews (29%), followed by bandicoot rats (14.3%) and black rats (8.5%) (Table 4).

**Table 4:** Molecular detection of *Orientia tsutsugamushi* in chigger mite pools in study of *Leptotrombidium imphalum* chiggers as vector for scrub typhus in human settlements, India, 2022–2023

Small mammal species	Prevalence in chigger pool, no. positive/no. tested (%)			
	Village center	Village edge	Field	Total
Black rat, *Rattus rattus*	8/61 (13.1)	3/76 (4)	9/98 (9.2)	20/235 (8.5)
Greater bandicoot rat, *Bandicota indica*	17/153 (11.1)	22/104 (21.2)	1/23 (4.4)	40/280 (14.3)
Indian gerbil, *Tatera indica*	0/0	0/3 (0)	0/9 (0)	0/12 (0)
Mouse, *Mus* spp.	0/0	0/9 (0)	0/8 (0)	0/17 (0)
Three-striped Indian palm squirrel, *Funambulus palmarum*	0/0	1/2 (50)	0/11 (0)	1/13 (7.7)
Asian house shrew, *Suncus murinus*	21/68 (30.9)	28/85 (32.9)	12/56 (21.4)	61/209 (29.2)
Asian gray mongoose, *Urva edwardsii*	0/0	0/0	0/1 (0)	0/1 (0)
Total	46/283 (16.3)	54/270 (20.0)	22/206 (10.7)	122/759 (16.1)

We observed considerable clustering of chigger pool positivity within villages (ICC = 0.09; p<0.001), but no evidence of correlation between trap success and pool positivity by habitat (r = −0.09 for village center, r = −0.08 for edge, and r = −0.07 for fields).

We performed real-time PCR on spleen tissue of 874 small mammals, of which 99 (11.3%, 95% CI 9.3%–13.6%) tested positive for *O. tsutsugamushi* (Table 5). The prevalence of infection in spleen tissue was higher in small mammals caught in the field (15.2%) than in those caught at the village edge (10.3%) and center (8.9%; p = 0.019 by score test for trend), in contrast to infection in chiggers by location. The high prevalence of *Orientia* infection in spleen tissue of shrews in the fields (31%) is a likely cause of the trend (Table 5). Conversely, despite high prevalence of infection in chigger pools from bandicoot rats, the prevalence of infection in spleen tissue was 2.5%.

**Table 5:** Molecular detection of *Orientia tsutsugamushi* in small mammal spleen tissue in study of *Leptotrombidium imphalum* chiggers as vector for scrub typhus in human settlements, India, 2022–2023

Small mammal species	Prevalence in spleen tissue, no. positive/no. tested (%)			
	Village center	Village edge	Field	Total
Black rat, *Rattus rattus*	9/79 (11.4)	9/82 (11)	9/112 (8)	27/273 (9.9)
Greater bandicoot rat, *Bandicota indica*	4/156 (2.6)	2/103 (1.9)	1/24 (4.2)	7/283 (2.5)
Indian gerbil, *Tatera indica*	0/0	1/3 (33.3)	3/9 (33.3)	4/12 (33.3)
Mouse, *Mus* spp.	0/1 (0)	0/0	0/21 (0)	0/22 (0)
Three-striped Indian palm squirrel, *Funambulus palmarum*	0/0	0/1 (0)	0/12 (0)	0/13 (0)
Asian house shrew, *Suncus murinus*	15/78 (19.2)	18/101 (17.8)	28/91 (30.8)	61/270 (22.6)
Asian gray mongoose, *Urva edwardsii*	0/0	0/0	0/1 (0)	0/1 (0)
Total	28/314 (8.9)	30/290 (10.3)	41/270 (15.2)	99/874 (11.3)

**Figure 4:**
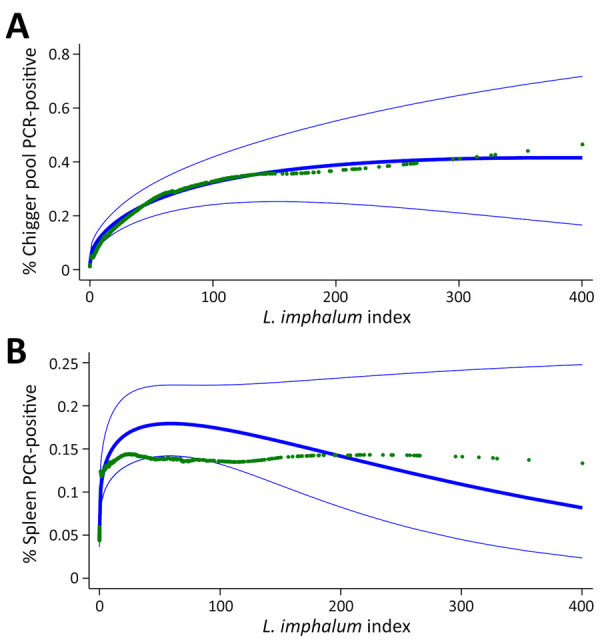
Association between *Leptotrombidium imphalum* index and the prevalence of *Orientia* infection in study of *L. imphalum* chiggers as vector for scrub typhus in human settlements, India, 2022–2023. A) Association between prevalence in the chigger pool collected from the host and chigger index. B) Association between prevalence determined from host spleen tissue and chigger index. Blue lines show predicted prevalence (thick) with 95% CI (thin). Green markers show locally weighted scatterplot smoothing of *Orientia* prevalence by *L. imphalum* index using a bandwidth of 0.4.

Adjusted for pool size, the prevalence of *Orientia* infection in chigger PCR pools increased with the estimated *L. imphalum* index on small mammals, leveling off at an index of ≈200 (Figure 4, panel A). By contrast, the association between the estimated *L. imphalum* index and prevalence of spleen tissue infection sharply plateaued at an *L. imphalum* index of ≈60 (Figure 4, panel B).

## Discussion

In this setting in South India where scrub typhus is a major public health problem (*13*), chigger numbers found on small mammals and the prevalence of *O. tsutsugamushi* infection in chiggers were higher within human settlements than in the land surrounding them. Several findings point to the peridomestic environment as a high-risk setting for infestation with *O. tsutsugamushi*–infected chigger mites. Trap success of small mammals, a proxy for density, was 4–5 times higher in village centers and edges than in the fields. Furthermore, chigger indices were higher in those locations, presumably because mite larvae more frequently encounter small mammals as suitable hosts (*21*), which they require to develop into the nymph and adult stages. *L. imphalum* chiggers, which have been implicated as a vector for scrub typhus in other foci across Asia (*22*,*23*), might be the dominant and perhaps only vector for scrub typhus in our setting. Of note, the estimated prevalence of *O. tsutsugamushi* infection in *L. imphalum* chiggers was more than twice as high in village center and edge than in the fields. Even when adjusting for pool size, a higher *L. imphalum* index, a potential marker of trombiculid mite density in the environment, was associated with a higher prevalence of infection in chigger pools (Figure 4, panel A). Our data suggest that, through mechanisms such as acquisition of infection from the host (*24*), cofeeding of chiggers (*25*,*26*), or horizontal transmission during other life stages, a high population density of *Leptotrombidium* mites may promote *Orientia* infection in the vector. Conclusive evidence only exists for vertical transmission (*24*,*27*).

A study in Yunnan, China, revealed close association between human habitats and *Leptotrombidium deliense* chiggers (*28*); that study and others from Thailand (*29*,*30*) found a low chigger species diversity in human settlements compared with less disturbed ecosystems. The occurrence of 1 *Leptotrombidium* chigger species, *L. imphalum*, in our study stands in contrast to earlier work from South India, which suggested a high *Leptotrombidium* chigger species diversity and an absence of *L. imphalum* chiggers in comparable settings (*31*–*33*). Future studies could include molecular barcoding to distinguish between morphologically similar species (*34*). In general, vector studies involving small mammals trapped in the peridomestic environment in South India have demonstrated high prevalence of *Leptotrombidium* infestation (*33*,*35*,*36*). Our study confirms that chigger mites regularly infest highly synanthropic small mammals, thus increasing the likelihood of infestation of humans.

As observed in earlier studies from India (*36*,*37*), *S. murinus* shrews carry large numbers of *Leptotrombidium* spp. chiggers (Table 2) and could play an important role in maintaining scrub typhus transmission. Bandicoot rats had lower *L. imphalum* indices than did the shrews but were highly synanthropic (Table 1) and likely to substantially contribute to *L. imphalum* density in villages. By contrast, the prevalence of infection in host spleen tissue plateaued at relatively low *L. imphalum* indices (Figure 4, panel B), suggesting that high exposure to *O. tsutsugamushi* might strengthen the immune response to the pathogen in hosts and enable them to rapidly clear the infection. The higher prevalence of *Orientia* infection in spleen tissue from hosts trapped in the field, compared with those from center and edge locations (Table 5), was driven by the high infection prevalence in house shrews, which might be less able to clear the infection than bandicoot rats.

Previous work has discussed causes of the strong seasonality of human scrub typhus in most settings (*4*,*38*). We found a strong correlation between human scrub typhus and trap success in the fields but not in village edge and center (Figure 1). The *L. imphalum* index correlated moderately with human scrub typhus, as in a study from Puducherry in South India (*37*). Small mammal trappings within villages increased in the dry season, inversely to human scrub typhus, suggesting that hosts may prefer the village environment in the dry season. Similarly, infestation with *E. bhattipadense*, the dominant chigger in the village center, correlated inversely with human scrub typhus. Trombiculid mites are able to complete their life cycle within burrows (*39*), perhaps independently of seasonal changes in temperature and humidity. Therefore, chigger numbers on small mammals may not accurately reflect human exposure to chiggers. Further, small mammals, in particular Asian house shrews (*40*), can have large home ranges (*4*) and can move between village and field (i.e., the trap location may be far away from the place where chiggers are acquired). Collecting questing chiggers in the environment may provide further insights, for example, by using the black plating method (*41*) in village center, edge, and field to estimate the risk for human exposure in each habitat, ideally complemented by human behavior studies with possible use of tracking devices. Such studies might also investigate the marked clustering of *Orientia* infection in chiggers we observed in our study and the observed variation in human scrub typhus risk and associated risk factors among study villages (*12*).

The seasonality of small mammal trappings might also be influenced by changes in food availability, especially in the dry season, when the baited traps could be more attractive. The bait used in this study, coconut with peanut butter, might attract host species differently. For example, Indian gerbils, common rodents in the Indian subcontinent that cause extensive crop damage (*42*), were caught in small numbers in this study. Given that it was the host with the highest *L. imphalum* index (Table 2), our results might have underestimated its role as a maintaining host of *L. imphalum* chiggers. Our study confirmed the utility of genetic barcoding in identifying common host species presenting with unusual morphology (Appendix Figure 3).

We based the species-specific chigger index on a relatively small sample of <20 mites per host undergoing morphologic identification. Furthermore, we included different mites for morphologic identification and *O. tsutsugamushi* detection using PCR, then estimated infection prevalence using regression models. Our approach does not rule out *Orientia* infection of a mite species at a very low prevalence. Ideally, the same chigger morphologically identified under the microscope would have been used for PCR, potentially following methods described previously (*43*), but that was not feasible given the large number of chiggers processed.

In conclusion, this study corroborates studies in South India suggesting human settlements as the setting in which most infestation with chigger mites infected with *O. tsutsugamushi* occurs (*10*–*12*,*35*). Our findings suggest a causal chain involving large numbers of small mammals in human settlements, enabling high trombiculid mite densities (*21*) and large populations of chigger mites, which might encourage the spread of *O. tsutsugamushi* within the vector population (*25*,*26*) and cause a high risk for human scrub typhus infection.

Appendix Additional information from study of *L. imphalum* chiggers as vector for scrub typhus in human settlements, India, 2022–2023.
