# Supplementary material for: Leptotrombidium imphalum Chiggers as Vector for Scrub Typhus in Human Settlements, India, 2022–2023
Source: Emerg Infect Dis. 2026 Feb;32(2):191–201. doi: 10.3201/eid3202.251170 (PMC12928224; doi:10.3201/eid3202.251170)
Supplement: Appendix — Additional information from study of L. imphalum chiggers as vector for scrub typhus in human settlements, India, 2022–2023. [file 25-1170-Techapp-s1.pdf]

Article DOI: https://doi.org/10.3201/eid3202.251170

EID cannot ensure accessibility for supplementary materials supplied by authors. Readers who have difficulty accessing supplementary content should contact the authors for assistance.

Leptotrombidium imphalum Chiggers as Vector for Scrub Typhus in Human Settlements, India, 2022–2023

Appendix

DNA Extraction from Chiggers and Small Mammal Spleen Tissue

Ethanol from Eppendorf tubes containing chiggers was carefully decanted and the remaining ethanol allowed to evaporate by placing the open tubes in a water bath at 70°C. This was followed by freeze-thawing using methods published by Silva de la Fuente et al. (*1*). Chiggers underwent three cycles of freezing in liquid nitrogen and thawing in a water bath at 70°C. Visual inspection of the chiggers under the light microscope suggested differences in tissue disruption by freeze-thawing among genera, with *Schoengastiella* maintaining a higher degree of tissue integrity than *Leptotrombidium* and *Ericotrombidium/Hypotrombidium* (Appendix Figure 2), possibly affecting DNA extraction success. To improve comparability of DNA extraction among different mite genera, the freeze-thawing process was followed by grinding of chiggers using a micropestle. DNA from chiggers was then extracted using the DNeasy Qiagen Blood and Tissue Kit (Hilden, Germany) with a final eluant of 30µl. DNA from spleen samples of small mammals (10mg) was extracted without further tissue disruption using the same kit, and with a final eluant of 200 µl.

Detection of *Orientia tsutsugamushi* in Chiggers and Host Spleen Tissue

The qPCR assay to detect *O. tsutsugamushi* targeted the 47-kDa protein gene following Jiang and colleagues (*2*). The final primer concentration (forward: OtsuFP630 - 5′-AAC TGA TTT TAT TCA AAC TAA TGC TGC T-3′; reverse: OtsuRP747 - 5′-TAT GCC TGA GTA AGA TAC RTG AAT RGA ATT-3′) was 0.1 µM. The final TaqMan probe concentration

(OtsuPR665 – 5′-6FAM-TGG GTA GCT TTG GTG GAC CGA TGT TTA ATC T-BHQ1–3′) was 0.2 μM. The final reaction volume of 25 μl contained 12.5 μl of Platinum Quantitative PCR SuperMix-UDG (Invitrogen), a total of 5mM Magnesium, and 5 μl (chiggers) or 1 μl (spleen tissue) of DNA template. The qPCR cycling conditions were: hold at 50°C for 2 min, hold at 95°C for 2 min, followed by 45 cycles of 95°C for 15 seconds (denaturation) and 60°C for 30 sec (Combined annealing/extension). A *ct* value of less than 39 was regarded as positive.

Molecular Barcoding of Small Mammals

The assay for molecular barcoding of small mammals targeted the COI gene as described previously (*3*), and used the primers Generalist-BatL5310 5′ CCTACTCRGCCATTTTACCTATG 3′ (forward) and Generalist-R6036R 5′ ACTTCTGGGTGTCCAAAGAATCA 3′ (reverse). The final reaction volume of 50μl contained 0.5μM of both primers, 2.5mM MgCl2, 0.2mM dNTP, 1U Taq polymerase (Invitrogen) and 5ul DNA template (DNA extraction from spleen tissue diluted 1:100). The PCR cycling conditions were: initial denaturation 4 min at 94°C, 40 cycles (30 s at 94°C, 30 s at 48°C, 60 s at 72°C), with a final extension of 10 min at 72°C. The PCR product underwent Sanger sequencing (forward only), followed by BLAST analysis.

Calculation of Prevalence of Infection in Individual *L. imphalum* Chiggers

Since only *L. imphalum* was reliably associated with positive PCR pools (Table 3), we calculated the prevalence of *Orientia* infection only for this species. We used a generic formula to calculate the prevalence of *Orientia* infection in *L. imphalum* chiggers from chigger pools proposed by Katholi et al. (*4*):

$P_{pool} = 1 - (1 - p)^s$ (*1*)

where P_{pool} is the proportion of pools that are positive for *O. tsutsugamushi*, p is the prevalence of *Orientia* infection in individual *L. imphalum* chiggers and s is the pool size. The formula assumes a uniform pool size. However, in our study, pool sizes varied between 1 and 20 chiggers. Further, the number of *L. imphalum* chiggers in a pool was extrapolated by multiplying the proportion of *L. imphalum* found on the microscopy slides prepared from chiggers from the same location (ear or leg) with the pool size of the Eppendorf. This estimated number of *L.*

imphalum chiggers in a pool was not necessarily a whole number. We applied two different methods to determine the prevalence of *Orientia* infection in *L. imphalum*: First, we applied generalized linear models using a binomial family and complementary log-log link with the log pool size as offset, excluding pools with an estimated number of *L. imphalum* of zero. The resulting prevalence of *Orientia* infection in *L. imphalum* was 3.6% (4.5% for village center, 4.2% for village edge and 1.9% for field).

To confirm the validity of these estimates, we used a second method in which the prevalence of infection was determined iteratively by listing all pools in a table, and summing up the expected probability of pool positivity from equation (*1*) (based on an assumed prevalence of infection in individual *L. imphalum*) and observed PCR positivity (Appendix Table 1). The prevalence of infection in individual *L. imphalum*, p, was iteratively modified until the observed number of PCR positive pools (N_{obs}) approximately equalled the predicted number of positive PCR pools (N_{pred}) based on equation (*1*). N_{pred} was calculated as the sum of the predicted probabilities p_{pool} of each pool (Appendix Table 1). Using this approach, the estimated prevalence of *Orientia* infection in *L. imphalum* was very similar to the complementary log-log models (3.6% overall, 4.5% village center, 4.1% village edge and 2.0% in the fields).

Description of *Walchia* sp., aff. *parvula*

Diagnosis. fPp = N/N/NNN; Ga = N; fCx = 1.1.1; fSt = 2.2; eyes 2 + 2; two genualae I; fD = 2H-6(7)-12-14–2-4-2; DS = 38–42; V = 48–52; NDV = 86–94. Differs from *W. parvula* by lesser AW (28–33 vs. 40), shorter setae (AL = 16–17 vs. 27; PL = 22–23 vs. 31), and by the presence of 6–7 setae in 1st posthumeral row vs. 8 (*5*).

Description of *Hypotrombidium* sp.

Diagnosis. SIF = 7B-B-3-2111.0000; fPp = B/B(b)/NNB; fCx = 1.1.1; fSt = 2.2; fD = 2H-8-6-6-4-2-2; DS = 30; V = 28; NDV = 58.

References

1. Silva de la Fuente MC, Pérez C, Martínez-Valdebenito C, Pérez R, Vial C, Stekolnikov A, et al. Eco-epidemiology of rodent-associated trombiculid mites and infection with *Orientia* spp. in southern

Chile. PLoS Negl Trop Dis. 2023;17:e0011051. PubMed https://doi.org/10.1371/journal.pntd.0011051

2. Jiang J, Chan TC, Temenak JJ, Dasch GA, Ching WM, Richards AL. Development of a quantitative real-time polymerase chain reaction assay specific for *Orientia tsutsugamushi*. Am J Trop Med Hyg. 2004;70:351–6. PubMed https://doi.org/10.4269/ajtmh.2004.70.351

3. Ceropath. Community ecology of rodents and their pathogens in southeast Asia. 2024 [cited]. www.ceropath.org

4. Katholi CR, Toé L, Merriweather A, Unnasch TR. Determining the prevalence of *Onchocerca volvulus* infection in vector populations by polymerase chain reaction screening of pools of black flies. J Infect Dis. 1995;172:1414–7. PubMed https://doi.org/10.1093/infdis/172.5.1414

5. Kudryashova NI. Chigger mites (Acariformes, Trombiculidae) of East Palaearctics. Moscow: KMK Scientific Press; 1998.

Appendix Table 1. Iterative estimation of *Orientia* infection prevalence in *L. imphalum.*

Pool serial number (*i*)	Pool size (expected number of *L. imphalum*)	Observed pool PCR result	p_{pool}
1	s_1	x_1	$1-(1-p)^{s_1}$
2	s_2	x_2	$1-(1-p)^{s_2}$
3	s_3	x_3	$1-(1-p)^{s_3}$
4	s_4	x_4	$1-(1-p)^{s_4}$
n	s_n	x_n	$1-(1-p)^{s_n}$
		$N_{obs}=\sum_{i=1}^{n} x_i$	$N_{pred}=\sum_{i=1}^{n} 1-(1-p)^{s_i}$

i, pool serial number; *s,* pool size; *x,* observed PCR result (0 = negative, 1 = positive); *p,* prevalence of *Orientia* infection in individual chiggers. *p* was iteratively modified until the observed number of PCR-positive pools (N_{obs}) approximately equalld the predicted number of positive PCR pools (N_{pred}).

Appendix Table 2. Measurements of 3 *Walchia* specimens.

AW	PW	SB	ASB	PSB	SD	AP	AL	PL	P-PL	pa	pm	pp	Ip
33	52	27	19	33	52	34	16	22	17	168	157	179	537
30	51	28	19	30	59	30	17	23	15	163	145	172	480
28	47	28	17	34	47	33	17	22	16	187	158	189	534

AL, length of anterolateral scutal setae; AP, distance from AL to PL; ASB, distance from anterior scutal margin to sensillary bases; AW, distance between Als; Ip, total leg length; pa, anterior leg length; PL, length of posterolateral scutal setae; pm, medial leg length; pp, posterior leg length; P-PL, distance from posterior scutal margin to PLs; PSB, distance from posterior scutal margin to sensillary bases; PW, distance between PLs; SB, distance between sensillary bases; SD, length of scutum.

Appendix Table 3. Measurements of three *Hypotrombidium* specimens.

AW	PW	SB	ASB	PSB	SD	P-PL	AP	AM	AL	PL	pa	pm	pp	Ip
58	64	23	23	11	33	4	27	33	39	49	303	272	302	877
61	71	25	21	12	35	5	26	28	35	47	306	264	303	873
63	75	24	25	11	35	6	26	33	36	46	295	258	295	848

AL, length of anterolateral scutal setae; AM, length of anteromedian scutal seta; AP, distance from AL to PL; ASB, distance from anterior scutal margin to sensillary bases; AW, distance between Als; Ip, total leg length; pa, anterior leg length; PL, length of posterolateral scutal setae; pm, medial leg length; pp, posterior leg length; P-PL, distance from posterior scutal margin to PLs; PSB, distance from posterior scutal margin to sensillary bases; PW, distance between PLs; SB, distance between sensillary bases; SD, length of scutum.

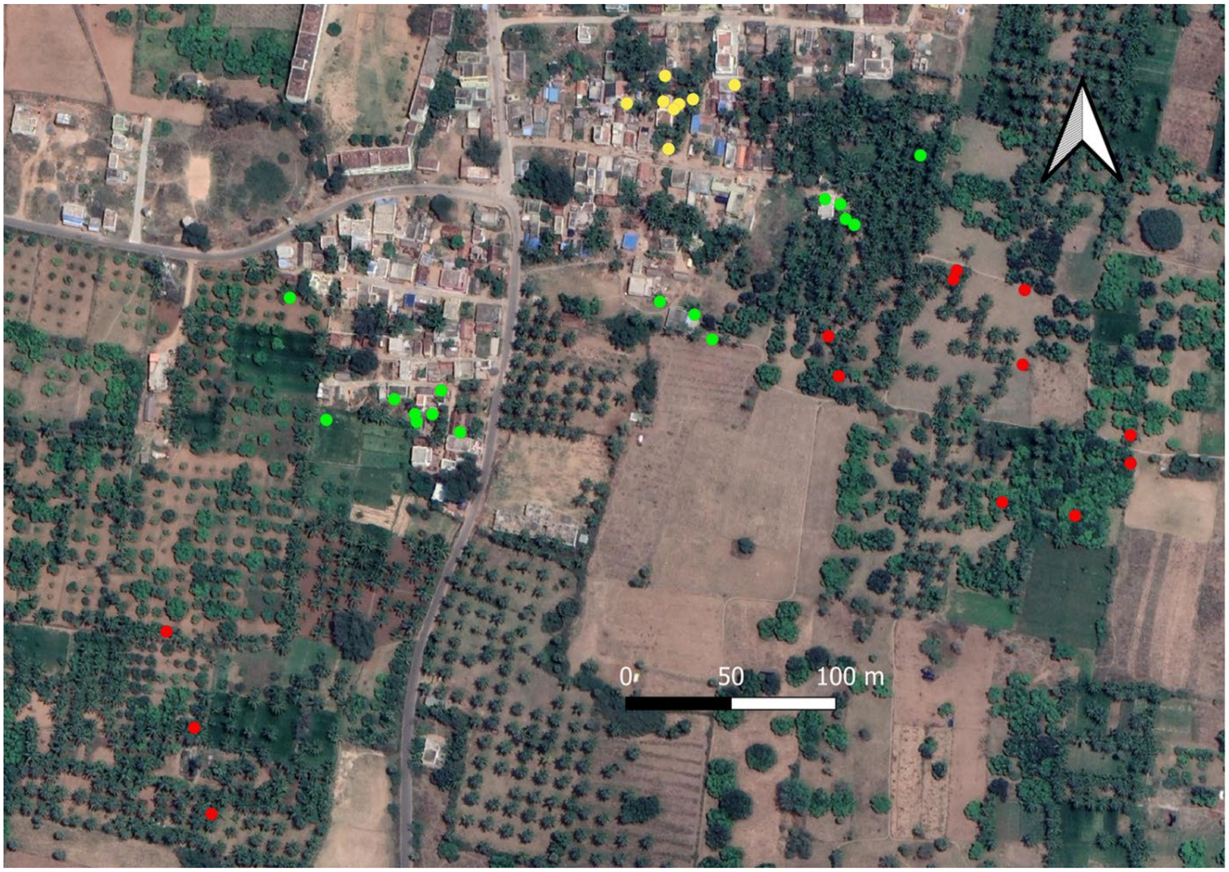

Appendix Figure 1. Example of trap locations in village center (yellow), village edge (green), and fields (red).

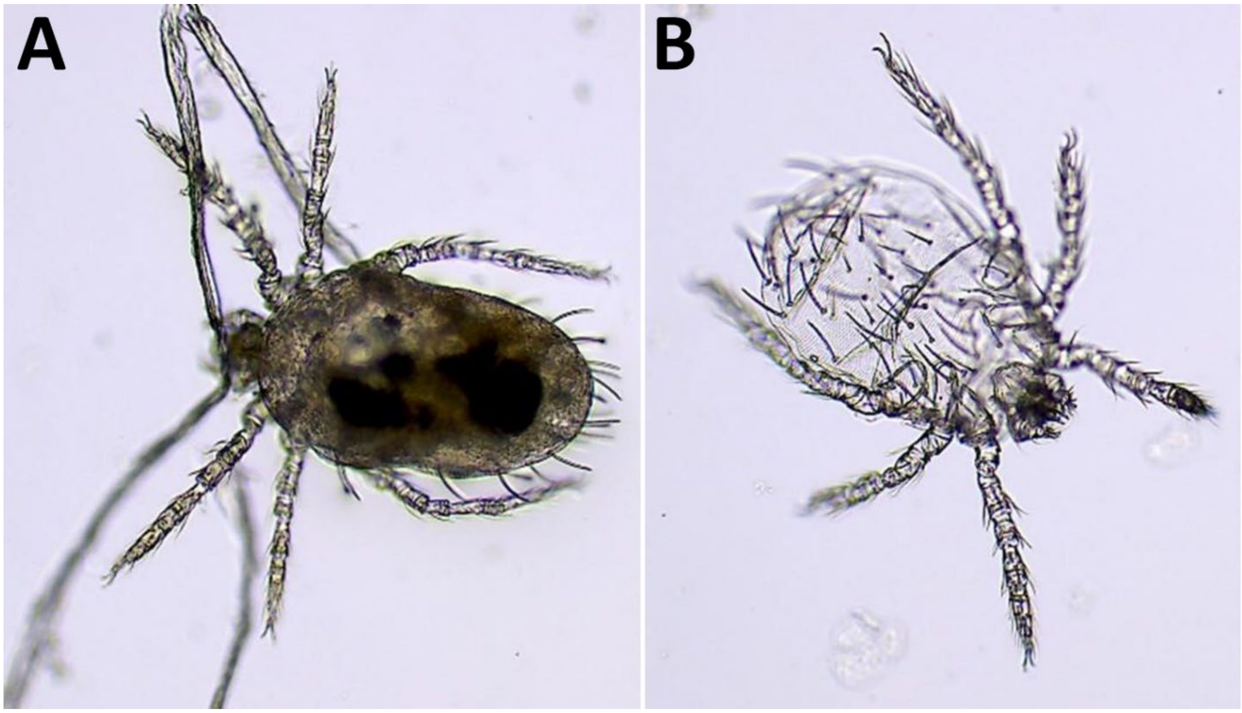

Appendix Figure 2. *Schoengastiella* sp. (left) and *Leptotrombidium* sp. (right) observed after three cycles of freeze-thawing. Note fully dissolved internal organs in *Leptotrombidium* sp. compared to the largely opaque *Schoengastiella* sp.

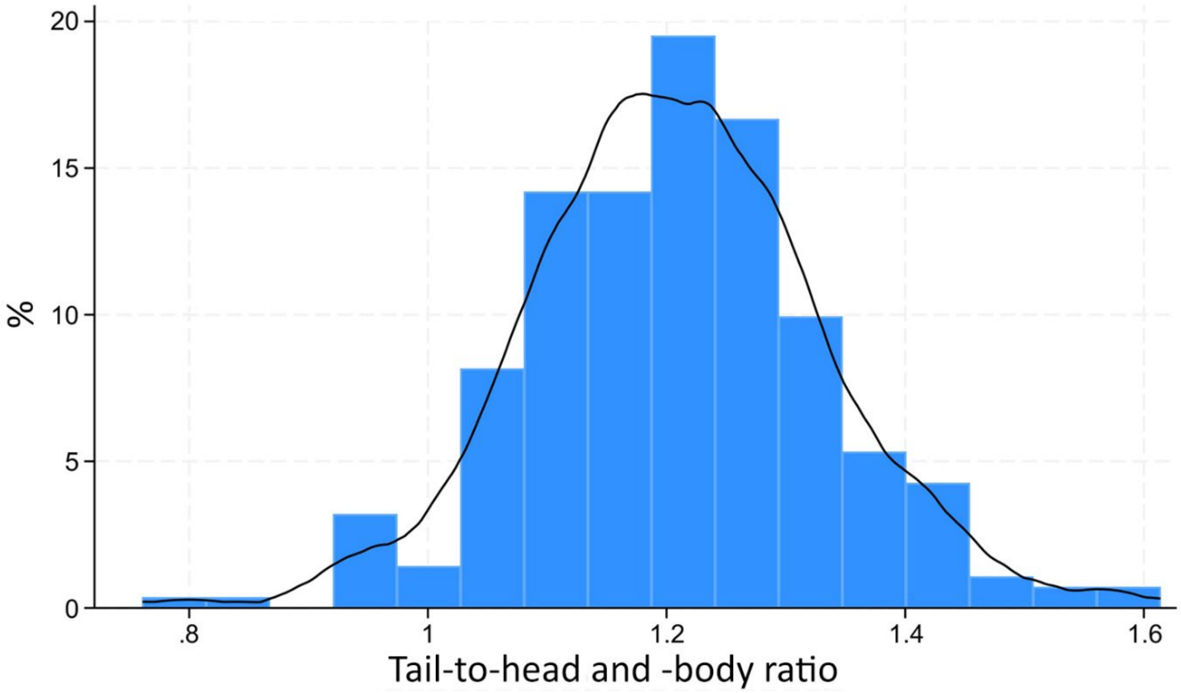

Appendix Figure 3. Histogram and Kernel density (half-width 0.03) of the Tail to Head+Body ratio of *Bandicota indica* (n = 284).

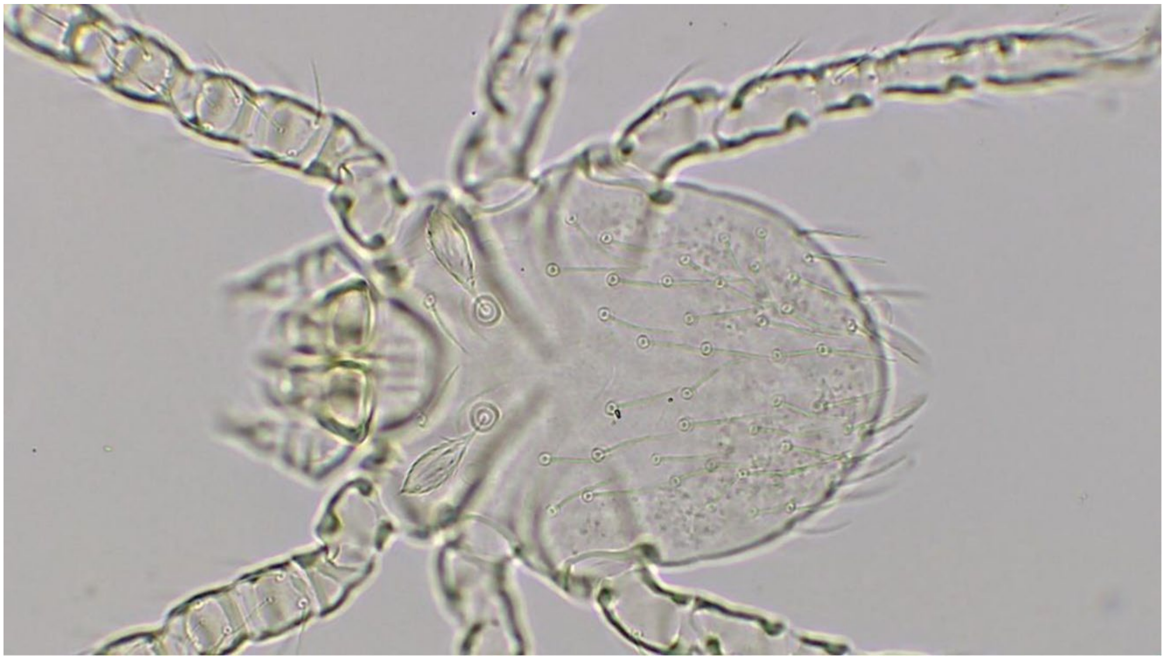

Appendix Figure 4. *Walchia* sp., dorsal aspect.

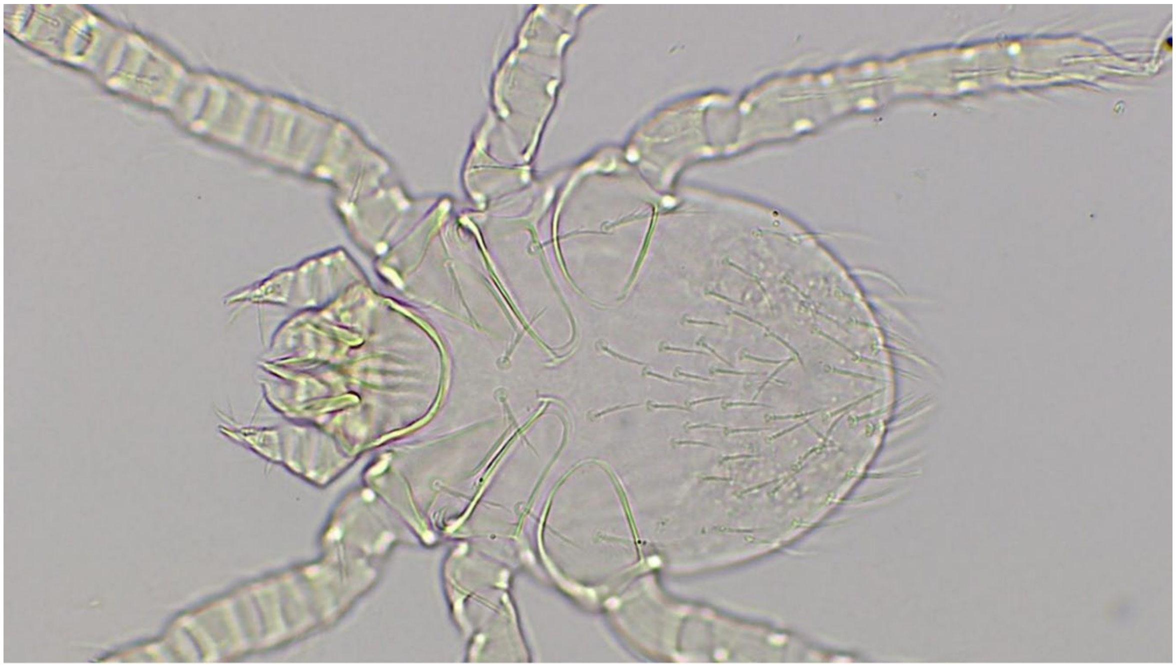

Appendix Figure 5. *Walchia* sp., ventral aspect.

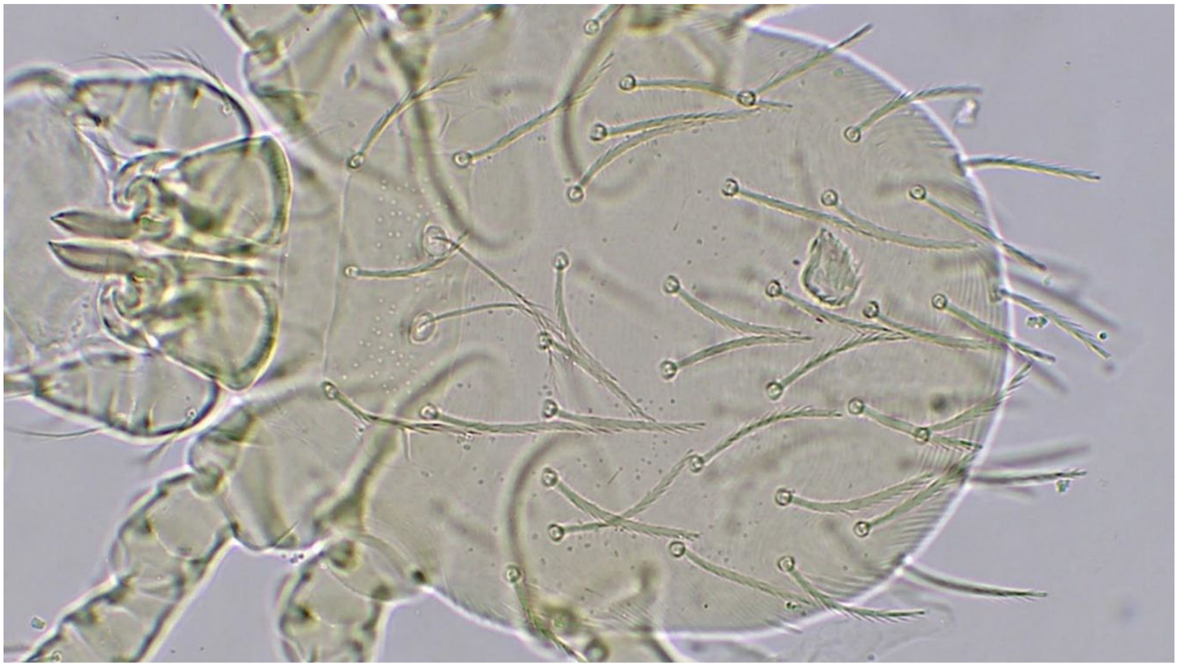

Appendix Figure 6. *Hypotrombidium* sp., dorsal aspect.

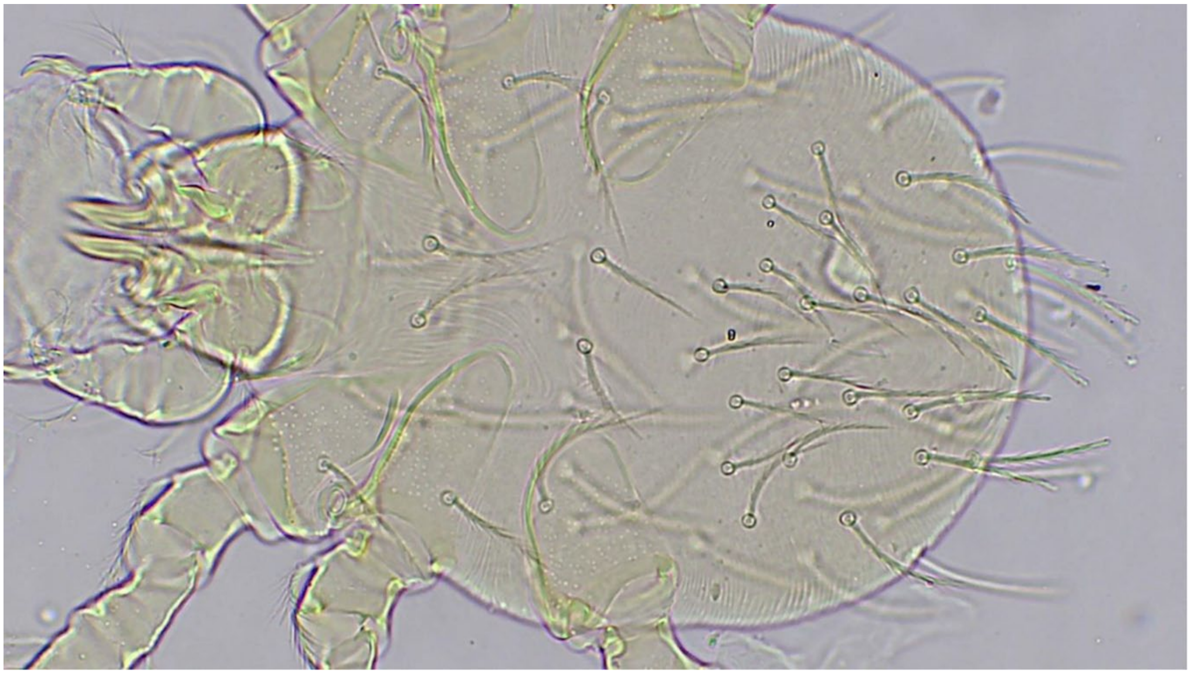

Appendix Figure 7. *Hypotrombidium* sp., ventral aspect.
